# Environmental Impact of High Concentration Nitrate Migration in Soil System Using HYDRUS Simulation

**DOI:** 10.3390/ijerph17093147

**Published:** 2020-04-30

**Authors:** Yuanyuan Zhang, Duujong Lee, Jing Ding, Jianfeng Lu

**Affiliations:** 1 School of Material Science and Engineering/School of Intelligent Systems Engineering, Sun Yat-Sen University, Guangzhou 510006, China; zhangyy228@mail2.sysu.edu.cn; 2 Department of Chemical Engineering, National Taiwan University, Taipei 10617, Taiwan; djlee@ntu.edu.tw

**Keywords:** nitrate migration, soil and groundwater, environmental impact, nitrate contamination time, numerical model

## Abstract

Nitrate is a promising heat transfer fluid in solar thermal power and nuclear power systems, but its leakage can cause serious environmental problems. The present paper investigates the deep and prolonged migration of high concentrations of nitrate into the soil system, and the associated diffusion range is studied to estimate and reduce the environmental pollution caused by nitrate leaks. The vertical nitrate contaminated range is mainly impacted by annual precipitation, soil properties and groundwater depth, while the horizontal contaminated range is mainly affected by the initial leakage area. During the process, the vertical contaminated range first continuously enlarges, and then decreases after a long time. The nitrate contaminant can exist and affect the environment for as long as 115–625 years, and the nitrate contamination time can be even longer in dry regions. Since nitrate diffuses more quickly in unsaturated regions rather than in saturated regions, the migration region and contaminated range both decrease as the groundwater depth is increased.

## 1. Introduction

Nitrate is widely used in many fields, including industry [1,2] and agriculture [3]. Low concentration nitrate migration has been considered in the research fields of agriculture and drinking water health [4]. Column leaching experiments are basically used to investigate the retention and leaching of nitrate in soil, and Pratiwi et al. [5] found that rice husk char in soil could adsorb ammonium and nitrate in solution. The nitrate leaching characteristics in different soils have been further investigated by considering plant species richness [6], biochar [7] and so on. Leimer et al. [6] measured the nitrate concentration in soil solution in grassland plant diversity between 2003 and 2006 in Germany, and stated that plant species richness decreased nitrate leaching from grassland. Kanthle et al. [7] analyzed nitrate leaching mitigation by comparing different kinds of biochar, and stated that there was significant reduction of nitrate leaching with increase in biochar application, and no interactive effect of soil organic carbon and biochar could be observed. As salt solution, the nitrate concentration in soil is remarkably affected by nitrogen fertilizers [8], irrigation [9], etc. Liu et al. [8] investigated the effects of nitrogen fertilizers on the growth and nitrate content of lettuce, and the results showed that the total nitrogen concentration in soil and nitrate concentration in lettuce increased as the amount of nitrogen fertilizer increased. Woli et al. [9] found that the nitrate concentration during the leaching process was larger with a smaller irrigation, a longer irrigation interval, a higher nitrogen rate, and a heavier soil. Additionally, nitrate concentration in water is influenced by the hydrogeological regime and other conditions. Parrino et al. [10] analyzed bromide and nitrate ions as indicators of groundwater quality in coastal territories. Leslie and Lyons [11] presented a 19-month time series measuring nitrate, chloride, and sulfate in local precipitation, reservoir and household tap waters in order to evaluate the anion concentrations. Schullehner et al. [12] assessed concentration variability in the distribution system of nitrate, nitrite, and ammonium, and found that nitrate concentrations at waterworks exits were highly correlated with nitrate concentrations within distribution nets or consumers’ taps.

Numerical models have been used to study nitrate diffusion phenomena in soil and groundwater, and the models include stochastic models [13] and deterministic models such as HYDRUS Program (HYDRUS), Finite Element subsurface FLOW system (FEFLOW), Leaching Estimation and Chemistry Model (LEACHM), and Root Zone Water Quality Model (RZWQM). Many researchers used HYDRUS-1D to study nitrate migration in soil. Tafteh and Sepaskhah [14] used the HYDRUS-1D model to evaluate drainage and nitrogen fertilizer movement in groundwater. Jiang et al. [15] proposed HYDRUS-1D to analyze the water flow and bacterial transport in the undistributed soil samples, and stated that a single-porosity flow model successfully simulated water flow under natural climatic conditions and spray irrigation. Hlavacikova et al. [16] presented an application of the simple methodology for deriving the water retention properties of stony soils, taking into account a correction for the soil stoniness, and used HYDRUS-1D to simulate the influence of bottom fluxes. Kodesova et al. [17] focused on the numerical modeling of fly-ash transport in three sands in the laboratory, and found that fly-ash mobility increased with increasing sand particle content. Wu [18] investigated nitrate transport in soil with two layers by HYDRUS-1D, but its migration depth was limited. Some other researchers used HYDRUS-2D and a numerical method coupled with HYDRUS. HYDRUS-2D model was calibrated and validated on measured data and implemented to estimate N leaching losses from the soil profile [19]. Pang et al. [20] proposed HYDRUS-2D to simulate water movement and solute transport in soil and groundwater with depth of 10 m and length of 68 m, and the model simulated the general trend of field observations for soil water content and potential well. Shelia et al. [21] coupled HYDRUS-1D with Decision Support System for Agrotechnology Transfer (DSSAT) to simulate soil water dynamics, crop growth and yield, and compared HYDRUS-1D simulations with those of the tipping bucket approach using the same datasets. Besides HYDRUS, other models as LEACHM, RZWQM are used to study nitrate migration. Asada et al. [22] modeled nitrogen leaching from andosols amended with different composted manures using LEACHM. Cameira et al. [23] used RZWQM to simulate the fate of nitrogen in the field soil–crop environment in the Mediterranean region.

High concentrations of nitrate leakage probably occur when huge containers containing nitrogen solution or nitrate molten salt are involved in accidents. These occurrences remarkably affect the soil environment and water quality. However, high concentration nitrate migration caused by molten salt leakage and its pollution impact in the soil system are rarely investigated. In this paper, deep and prolonged nitrate migration in the soil system is first simulated by HYDRUS. Based on the numerical model, the unsteady nitrate diffusion performance in soil and groundwater can be studied, and the effects of precipitation, groundwater depth and soil material can be further considered in the study nitrate transport and pollution characteristics. The main aim of this article is to find the migration phenomena and environmental impact of high concentrations of nitrate in soil and groundwater systems. In addition, the nitrate contaminated range and time can be estimated, meaning that nitrate pollution can be better prevented and controlled.

## 2. Physical Model and Experimental Validation

### 2.1. System Description

**Figure 1:**
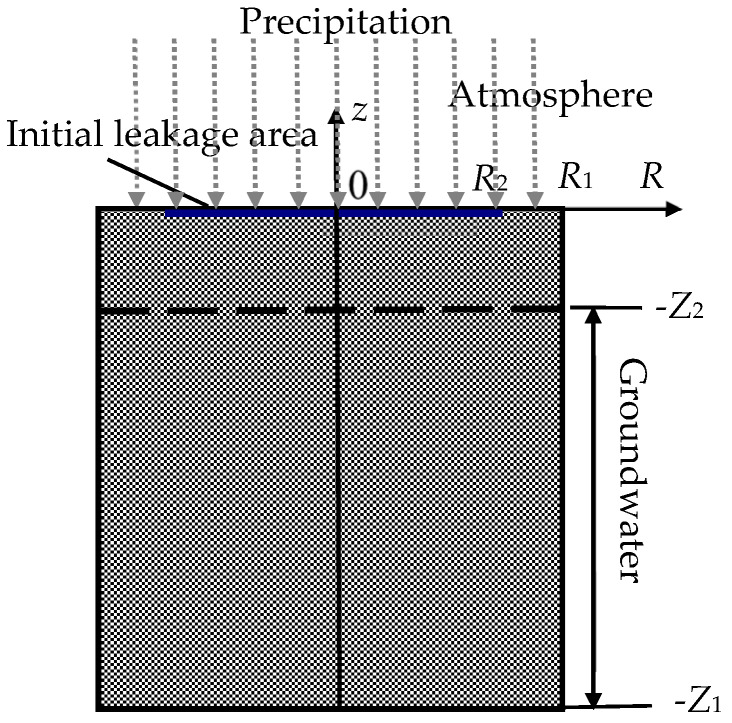
Nitrate migration in soil and groundwater.

In this paper, deep and prolonged migration of nitrate in soil and groundwater is simulated as in Figure 1. The radius and height of soil column are respectively *R_1_* and *Z_1_*. The upper surface of soil is open to the atmosphere, and the depth of groundwater surface is *Z_2_*. After molten salt leaks, molten salt expands above the soil as a thin layer [24], and the initial leakage radius is *R_2_*. The local precipitation flows from the top, and then the soil is infiltrated by rainfall, while molten salt diffuses inside the soil and groundwater.

### 2.2. Governing Equations

The water flow and solute transport process in soil and groundwater can be simulated and analyzed by the numerical simulation package HYDRUS [25]. The present article mainly investigated nitrate migration in soil systems, and four main assumptions are proposed as follows:

1. Soil is unexpansive, and its structure remains unchanged during the nitrate migration process;
2. No air phase exists in the liquid flow, and the gas pressure potential is zero. Compared with water pressure potential, gas pressure potential can be ignored for very low densities;
3. No chemical reaction is considered. A molten salt system is normally found in sterilized soil instead of microbially active soil, so reactions such as nitrification and denitrification can be ignored for the lack of microorganisms [26].
4. Since natural conditions are too complex, unnatural conditions are used without taking into account the hydrogeological regime, the number of aquifers, the presence of hydrogeological windows, and so on.

The water flow in soil is directly calculated by Darcy’s equation. The governing equation of water content is calculated by the following modified form of Richard’s equation [27]:

(1) $$\frac{\partial \theta }{\partial t}=\frac{\partial }{\partial x_{i}}\left[K\left(K_{ij}\frac{\partial h}{\partial x_{j}}+K_{iz}\right)\right]-Q$$

where *θ* is the volumetric water content, *t* is time, *h* is the pressure head, *x_i_* and *x_j_* are the spatial coordinates, *K* is unsaturated hydraulic conductivity function, *K_ij_* and *K_iz_* are components of dimensionless anisotropy tensor, and the sink term *Q* represents the volume of water removed per unit time from a unit volume of soil due to water uptake. For an isotropic medium [28], the diagonal entry *K_ij_* = 1, and the off-diagonal *K_ij_* = 0. *K_iz_* = cos α, where *α* is the angle between the flow direction and the vertical direction.

Van Genuchten [29] used the statistical pore-size distribution model from Mualem [30] to obtain a predictive equation for the unsaturated hydraulic conductivity function in terms of soil water retention parameters, and the unsaturated hydraulic conductivity function can be calculated as:

(2) $$K=K_{s}S_{e}{}^{l}{\left[1-{\left(1-S_{e}{}^{1/m}\right)}^{m}\right]}^{2}$$

where *K_s_* is the saturated hydraulic conductivity, *K_s,_ l* and *m* are parameters predicted by HYDRUS, and *S_e_* is effective water content. The pore connectivity parameter in the hydraulic conductivity function is estimated to be 0.5, as an average of many soils.

By referring to Richard’s Equation [27], the classical solute transport equation is derived by a mass conservation equation, and the non-equilibrium equation is taken as:

(3) $$\frac{\partial }{\partial t}(\theta C+\rho s)=\nabla (\theta D\nabla C)-\nabla (\theta uC)+G$$

where *C* and *s* are solute concentration in the liquid and solid phases, respectively, *D* is the dispersion coefficient tensor for the liquid phase, and *ρ* is the bulk density of soil, *u* is pore-water velocity, *G* is source sink term caused by a chemical reaction in the fluid and soil.

In the present article, fluid is assumed to be incompressible, and there is little reaction/sorption in the solid phase, so *s* = *G* = 0. In addition, the non-equilibrium equation from Equation (3) can be simplified as:

(4) $$\frac{\partial }{\partial t}(\theta C)=\nabla (\theta D\nabla C)-\nabla (\theta uC)$$

From Equations (1)–(4), the solute transport in soil and groundwater can be obtained by HYDRUS.

### 2.3. Calculation Conditions

According to previous research, the initial leakage radius of molten salt [24] is mainly determined by leakage conditions such as salt temperature, leakage time and soil parameters, and the nitrate diffusion range in soil is mainly affected by the initial leakage radius, soil parameters, annual precipitation and other conditions [28]. The size of calculation zone should be large enough for the nitrate diffusion process. For a typical deep and prolonged nitrate migration system in this article, the initial leakage radius of molten salt is assumed to be *R_2_* = 3.5 m, and then *R*_1_ = 5 m. In the vertical direction, the depth of the groundwater is *Z_2_* = 6 m, and *Z*_1_ = 50–150 m.

In the present simulation, three soils in typical regions (Dunhuang, Inner Mongolia and Beijing) are used, and their basic properties are shown in Table 1. The physical composition of soil is measured by pipette method based on soil particle analysis and the measurement device [28], and the soil particle classification standards are as follows: sand (0.05–1 mm), silt (0.005–0.05 mm) and clay (<0.005 mm). According to international soil texture classification, SOIL I, II and III are respectively sandy soil, silt soil and clay. The bulk density of soil in Table 1 is measured using the pycnometer test method. The true density of soil is measured by the pycnometer test method after drying, degassing and water injection processes. The porosity of the soil is calculated by bulk density and true density, but it is not used in HYDRUS. The salt diffusivity in water is correlated by a nitrate leaching experiment [28], and *D* = 0.1–5.1 cm^2^/d for SOILs III-I. The saturated hydraulic conductivity *K_s_* is estimated by the soil parameters in HYDRUS, and *K_s_* = 3.3–387.4 cm^2^/d for SOILs III-I.

The pressure head inside soil changes with the rainfall, and the initial pressure head inside the soil can be assumed as:

(5) $$h\left(z,t=0\right)=-z-Z_{2}$$

The boundary condition for the side and bottom soil columns is calculated by the Cauchy type boundary condition as follows:

(6) $$-\theta D\nabla C=\theta u\left(C_{in}-C\right)$$

where *C_in_* is the nitrate concentration of the incoming fluid.

The boundary condition for column top is calculated by precipitation data as:

(7) $$u\theta =\frac{P}{\Delta t}$$

where *P* is the monthly precipitation and Δ*t* is the time interval of one month.

**Figure 2:**
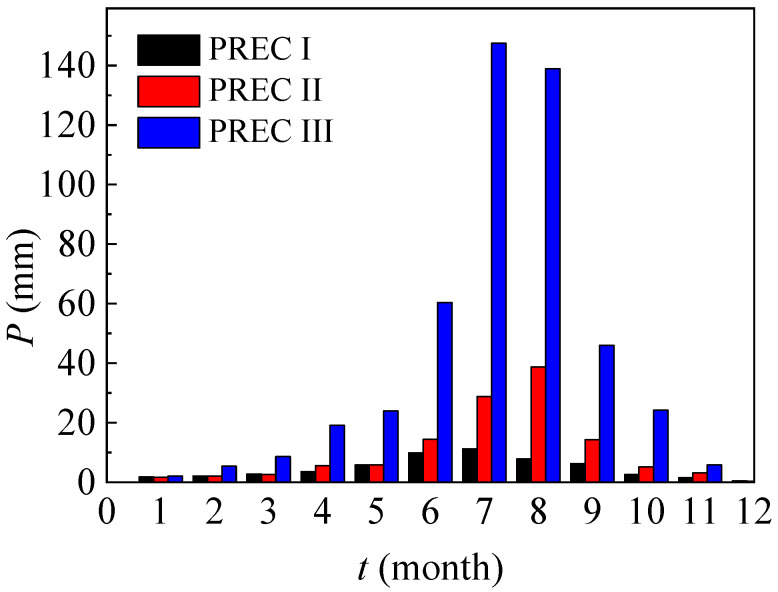
Monthly precipitation in a typical year (typical regions for PREC I, II and III are Dunhuang, Inner Mongolia and Beijing, and PREC means precipitation).

In this article, monthly precipitation data in three typical regions (Dunhuang, Inner Mongolia and Beijing) are presented in Figure 2, and their annual precipitation values are, respectively, 55.9 mm, 122.9 mm and 483.4 mm.

At the initial time, the leaked nitrate dissolves in water near the upper surface of the soil, and an initial salt solution layer exists as:

(8) $$C\left(R,z,t=0\right)=C_{0}\;\mathrm{for}\;0\leq R\leq R_{2},-Z_{3}\leq Z\leq 0$$

In the present article, the initial nitrate concentration is the saturation concentration in water solution *C_0_* = 651 mg/mL, and the depth of the initial salt solution layer is *Z_3_* = 0.1 m.

### 2.4. Model Validation and Discussion

**Figure 3:**
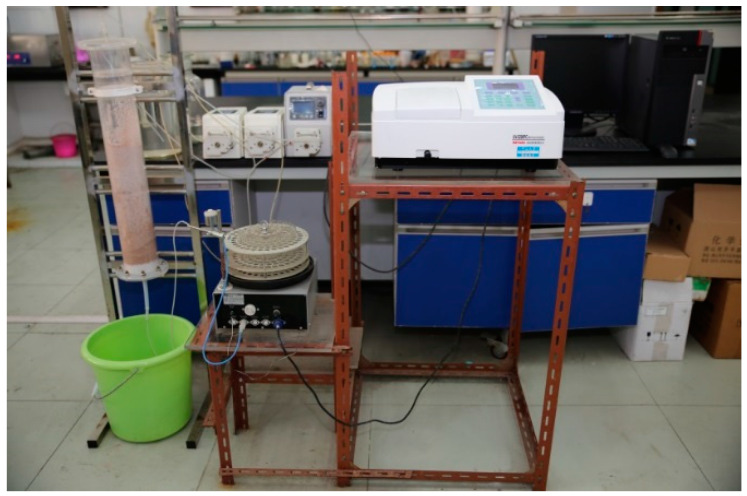
Nitrate migration experimental system.

In order to validate the numerical model for high concentration nitrate migration, a small scale experimental system has been established [28], as illustrated in Figure 3. The cylinder, which is 700 mm in height and 110 mm in inner diameter, is made of plexiglass, and SOIL I is packed inside the cylinder. In the present experiment, water drops from the shower are used to simulate rainfall. Before the experiment, pure water drips into the cylinder. In the first 40 min, a water solution with 100 g/L nitrate drips into the soil and, after that, pure water drips into the soil. The nitrate concentration of the outlet solution is analyzed using an ultraviolet spectrophotometer UV6000-PC (Shanghai Metash instruments Co. Ltd., Shanghai, China).

**Figure 4:**
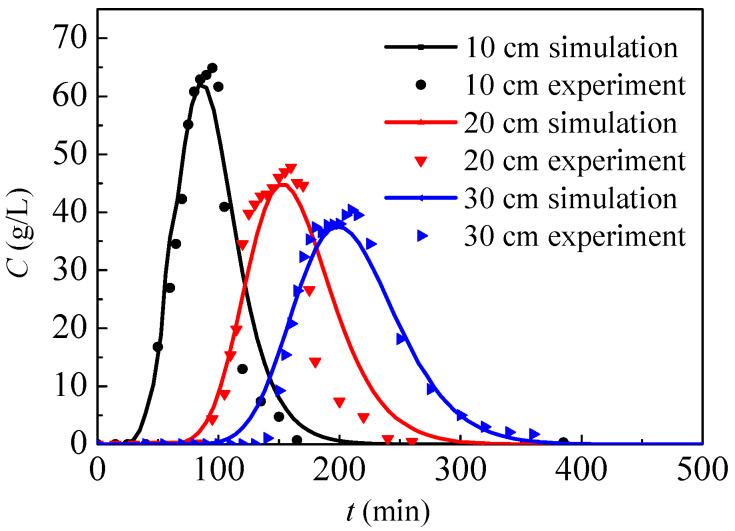
Nitrate concentration of the outlet solution from simulations and experiments.

By using the present numerical model and the associated parameters of the experiment, the nitrate concentration of the outlet solution can be calculated. Figure 4 presents the nitrate concentration of the outlet solution from simulations and experiments, where the heights of soil columns are, respectively, 10 cm, 20 cm and 30 cm. In general, the simulation results show good agreement with the experimental data, and the nitrate concentration in the outlet solution first increases to its maximum and then gradually decreases. In conclusion, the present physical model can be used to simulate high concentration nitrate migration in soil. Before the experiment, pure water flows through the soil. After the nitrate solution drips from inlet, the outlet nitrate concentration will gradually increase after a time-delay as nitrate diffuses from the inlet to outlet, and the time-delay will increase with the rising cylinder height. Because the nitrate solution only drips for 40 min, the outlet nitrate concentration will increase up to a peak, followed by a decrease. According to model assumptions, only nitrate diffusion without a chemical reaction is simulated by the present model. If nitrate reacts with soil during the diffusion process, the nitrate concentration drops and then the contaminant range probably decreases.

**Figure 5:**
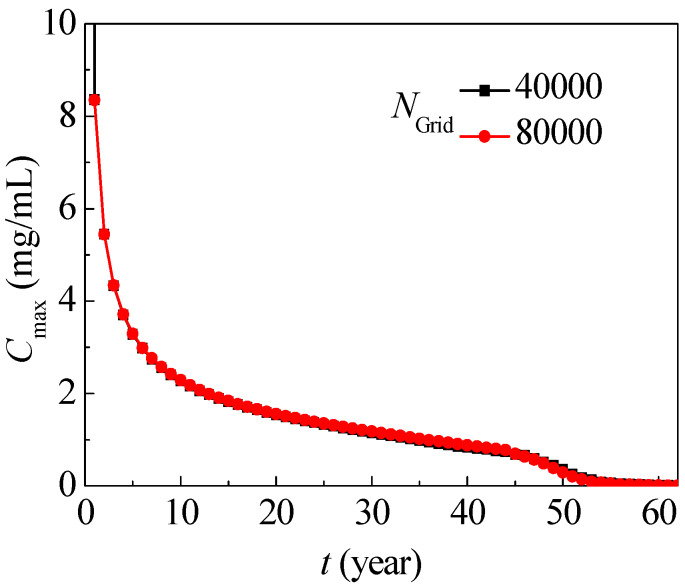
Maximum nitrate concentration evolution with different grids.

Calculations of typical nitrate migration (SOIL I, PREC III) in Figure 1 with 20,000, 40,000 and 80,000 elements yielding similar results, of which the mean deviation is below 0.08, as in Figure 5, and the grid with 40,000 elements is used with grid independence. The time step is 0.0001. The maximum allowable nitrate concentration is 0.0113 mg/mL according to the World Health Organization (WHO). According to the Chinese hygienic standard for drinking water (GB5749-2006), the permissible nitrate concentration must not exceed 0.01 mg/mL, and this value is used to judge whether the target zone is contaminated in this simulation.

## 3. Basic Nitrate Migration Performance in Soil System

**Figure 6:**
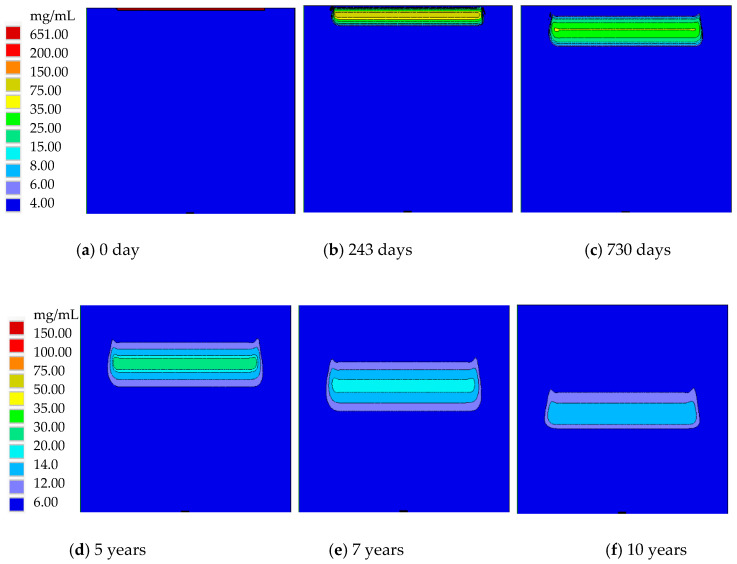
The nitrate concentration distribution in Dunhuang (SOIL I, PREC I).

In this section, basic nitrate transport in Dunhuang (SOIL I, PREC I) is investigated. After the nitrate solution diffuses into the soil and groundwater, the nitrate concentration quickly changes. Figure 6 presents the nitrate concentration distribution in Dunhuang. Because of rainfall in the vertical direction, nitrate mainly diffuses in the vertical direction, while it slightly diffuses in the horizontal direction. At the initial time, a very thin layer with a high nitrate concentration exists near the soil surface. After that, nitrate gradually migrates into the soil and groundwater, and its maximum nitrate concentration remarkably drops. The high concentration nitrate region with *C* > 2 mg/mL first expands for seven years and then shrinks after that.

**Figure 7:**
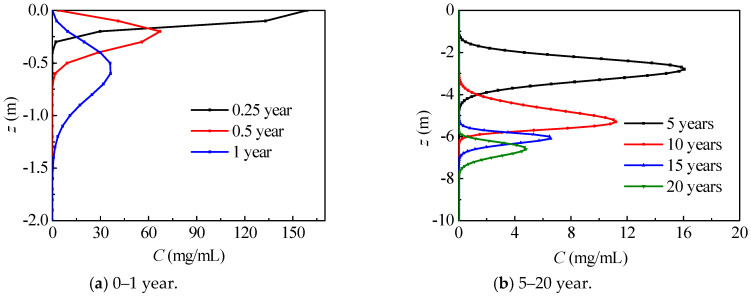
The axial nitrate concentration distribution in Dunhuang (SOIL I, PREC I).

Figure 7 describes the axial nitrate concentration distribution in Dunhuang. During the nitrate migration process, the maximum nitrate concentration remarkably drops, while the range with high concentration nitrate first enlarges and then shrinks. Most of the time (*t* > 0.5 year), nitrate concentration first increases and then decreases in the vertical direction. In the first 5 years, nitrate migrates within unsaturated region (*z* > −6.00 m), and maximum nitrate concentration remarkably drops to 16.1 mg/mL. During the 10–15 year period, nitrate migrates from the unsaturated region to the saturated region (*z* < −6.00 m), and maximum nitrate concentration remarkably drops from 11.2 mg/mL to 6.5 mg/mL for the water content increment. After 15 years, nitrate migrates inside the saturated region, and the maximum nitrate concentration slowly drops.

**Figure 8:**
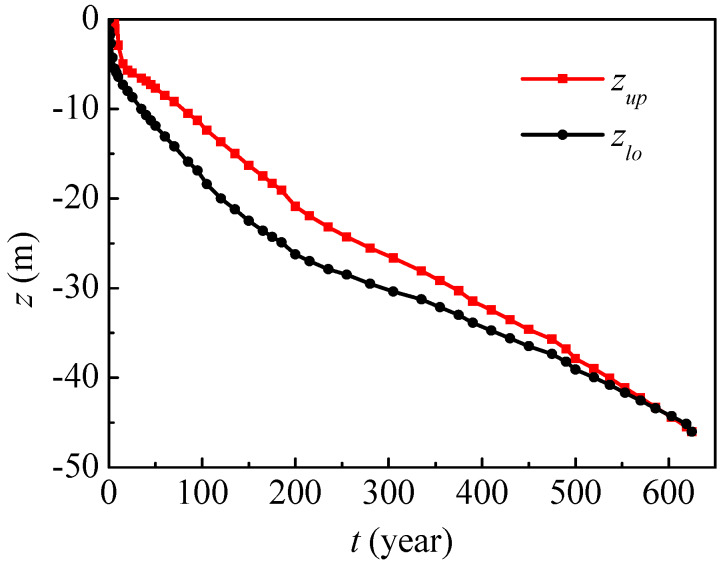
Nitrate contaminated range evolution in Dunhuang (SOIL I, PREC I).

Figure 8 presents vertical nitrate contaminated range evolution in Dunhuang, where *z_up_* and *z_lo_*, respectively, denote the upper and lower boundaries of the contaminated range (nitrate concentration C > 0.01 mg/mL). In the first half of the year, the contaminated upper boundary is the ground surface for nitrate concentrations higher than 0.01 mg/mL, while the contaminated lower boundary gradually decreases to −1.3 m. After half a year, the contaminated upper boundary begins to drop, and the lower boundary drops more quickly. In the eighth year, nitrate begins to migrate from the unsaturated region to the saturated region. In the 120th year, the contaminated upper and lower boundaries will respectively decrease to −13.7 m and −20.0 m, and the vertical contaminated range will enlarge to its maximum of 6.3 m. After that, the vertical contaminated range will gradually decrease. After 625 years, the maximum nitrate concentration will be less than 0.01 mg/mL, and this time is referred to as the nitrate contamination time.

## 4. Nitrate Migration under Different Conditions

### 4.1. Different Places

**Figure 9:**
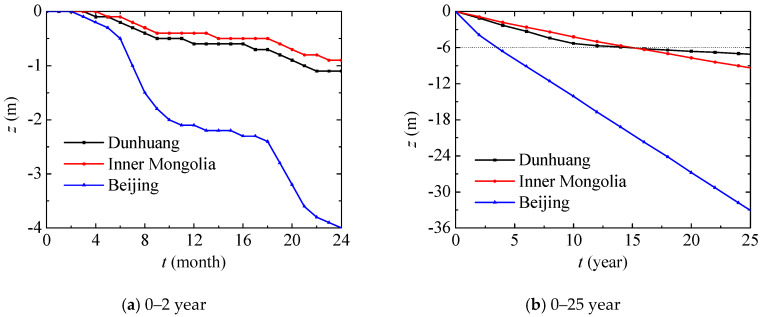
Vertical position evolution with maximum nitrate concentration in different places.

In this section, nitrate transport in different places including Dunhuang (SOIL I, PREC I), Inner Mongolia (SOIL II, PREC II) and Beijing (SOIL III, PREC III) will be compared. The evolutions of the vertical position with the maximum nitrate concentration in different places are presented in Figure 9, and they show a similar tendency. During the 0–5 month period, the vertical position, alongside the maximum nitrate concentration slowly decreases when there is very little monthly precipitation. During the 6–9 month period, the monthly precipitation is large, and the vertical position, alongside the maximum nitrate concentration, can quickly decrease. During the 9–17 month period, nitrate diffuses slowly due to the small amount of monthly precipitation. During the 18–21 month period, nitrate diffusion is similar to that during the 6–9 month period or one year ago. In general, nitrate diffuses in a cycle of one year due to precipitation. In Beijing, nitrate penetrates more deeply for high annual precipitation. Compared with Dunhuang, nitrate in Inner Mongolia first penetrates slowly due to the lower content of sand in the unsaturated region, and then penetrates quickly due to the higher annual precipitation in the saturated region after 15 years.

**Figure 10:**
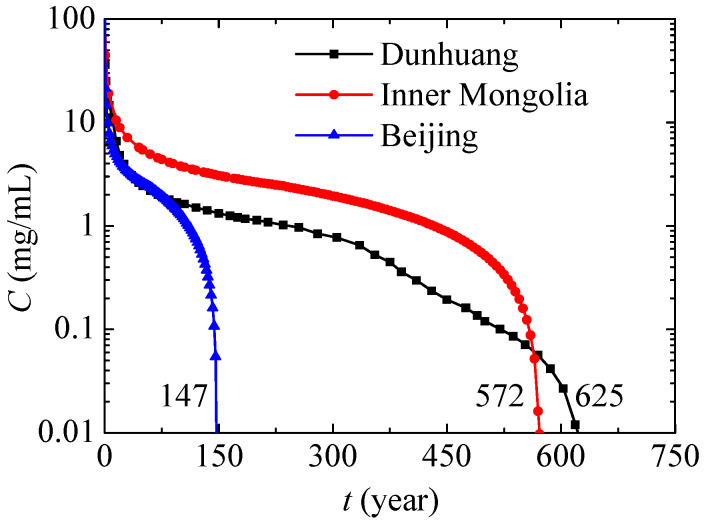
Maximum nitrate concentration variations in different places.

Figure 10 presents the maximum nitrate concentration variations in different places. In Beijing, the maximum nitrate concentration decreases most quickly for high annual precipitation. In Inner Mongolia, the content of sand in soil is close to that of Beijing, so its tendency in terms of maximum nitrate concentration variation is also similar, while the maximum nitrate concentration decreases more slowly due to lower annual precipitation. Compared with Inner Mongolia, Dunhuang has lower annual precipitation and higher content of sand, and because of this the associated nitrate concentration first decreases more quickly and then decreases more slowly. Under the present conditions, the nitrate contamination times of Beijing, Inner Mongolia and Dunhuang are respectively 147 years, 572 years and 625 years. In conclusion, nitrate will exist and affect the environment for a very long time especially in dry regions or in soil with a low content of sand, and the leaked nitrate should be removed as soon as possible.

### 4.2. Different Annual Precipitation

**Figure 11:**
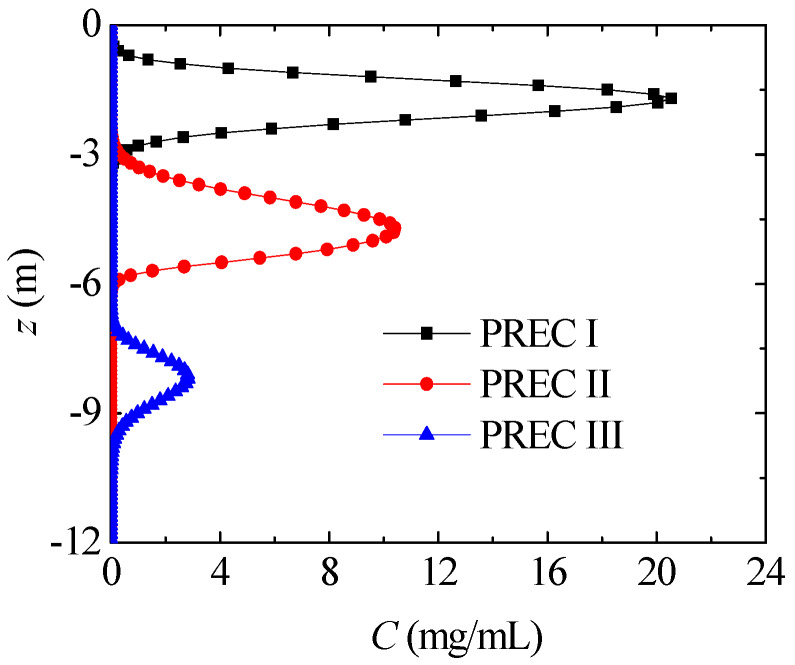
Nitrate concentration distribution with different annual precipitations (SOIL I, t = 6 years).

In this section, nitrate transport in SOIL I is compared with different annual precipitations. Figure 11 presents the axial nitrate concentration distribution after six years with different annual precipitations. As annual precipitation rises, the nitrate can permeate into deeper levels. Under low annual precipitation, as in PREC I and II, the nitrate mainly migrates within the unsaturated region (z > −6 m) after six years, and its concentration is still very high. Under high annual precipitation, as in PREC III, nitrate migrates into the saturated region after six years, and its concentration remarkably drops due to the higher water content.

**Figure 12:**
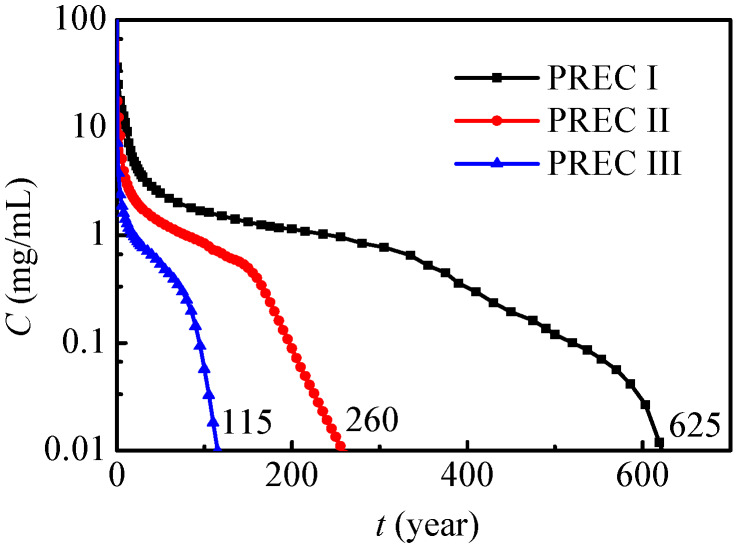
Maximum concentration variations under different annual precipitations (SOIL I).

When the nitrate leaks into the soil and groundwater, how the maximum nitrate concentration reduces to 0.01 mg/mL needs to be further analyzed. Figure 12 presents the maximum nitrate concentration variations under different annual precipitations. In general, the maximum nitrate concentration has a similar tendency under different levels of annual precipitation, and it gradually decreases to 0.01 mg/mL. As annual precipitation increases, the maximum nitrate concentration decreases, and the nitrate contamination time can be remarkably reduced at the same time. Under the present conditions, the nitrate contamination times under PREC I, II and III are respectively 115 years, 260 years and 625 years. In conclusion, nitrate will exist and affect the environment for a very long time, especially in dry regions, and the leaked nitrate should be removed as soon as possible. The results from Wang et al. [31] showed that nitrate leaching in the rainy season was higher than in dry and normal years, and this conclusion fits very well with the present results in Figure 12.

### 4.3. Different Soil

**Figure 13:**
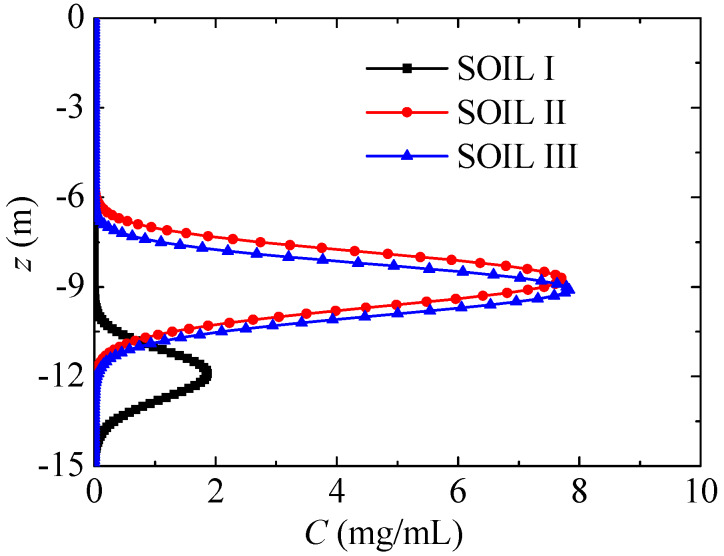
Nitrate concentration distribution in different soils (PREC III, t = 6 years).

In this section, nitrate transport with PREC I is compared in different soils. Figure 13 presents the nitrate concentration distribution in different soils, where t = 6 years. Compared with SOILs II and III, nitrate in SOIL I diffuses more deeply, and its maximum nitrate concentration is remarkably lower. In SOILs II and III, the percent of sand has little difference, and nitrate transport is also very similar. As a result, soils with large percentages of sand have more gaps, which are larger and show better permeability, and the nitrate diffuses more quickly. Kodesova et al. [17] stated that fly-ash mobility increased with increasing sand particles, and nitrate diffusion in different soils in Figure 13 shows a similar tendency.

**Figure 14:**
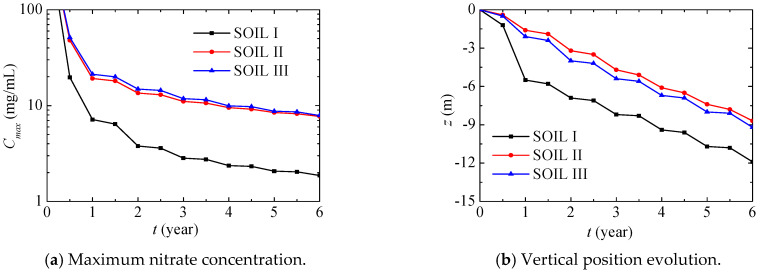
Maximum nitrate concentration and its vertical position evolution in different soils (PREC III).

Figure 14 further presents the maximum nitrate concentration and its vertical position evolution in different soils during the first six years. The maximum nitrate concentration quickly decreases in the first year, and then it gradually decreases after this, while the associated vertical position gradually decreases. Compared with SOILs II and III, the maximum nitrate concentration in SOIL I decreases more quickly, and its vertical position is remarkably lower.

### 4.4. Different Groundwater Depth

**Figure 15:**
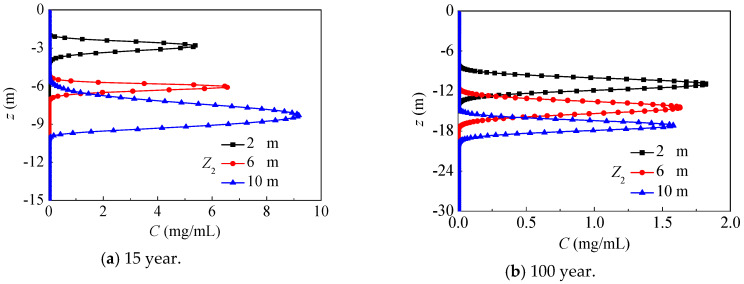
The nitrate concentration distribution under groundwater depths (SOIL I, PREC I).

In this section, different groundwater depths are considered with SOIL I and PREC I. Figure 15 presents the axial nitrate concentration distribution after 15 and 100 years, where the groundwater depth is, respectively, 2 m, 6 m and 10 m. For larger groundwater depths, the unsaturated region is larger, and the nitrate can migrate quickly and more deeply in this region, while the nitrate concentration in the unsaturated region is remarkably higher for lower water content. At 15 years, the maximum nitrate concentration and its vertical depth both increase with the rising groundwater depth, because nitrate mainly migrates in unsaturated regions for groundwater depths of 10 m, while it mainly migrates in saturated region for groundwater depths of 2 m. After 100 years, nitrate mainly migrates in saturated regions, and the maximum nitrate concentration gradually drops with a rising groundwater depth, while its position decreases from −10.9 m to −17.2 m.

**Figure 16:**
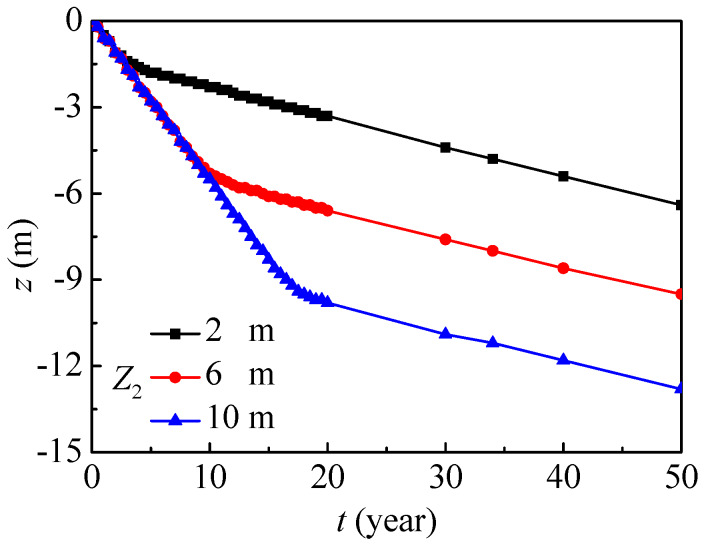
The vertical position evolution with maximum nitrate concentration under groundwater depths (SOIL I, PREC I).

Figure 16 further presents the vertical position evolutions with the maximum nitrate concentration. In general, the vertical position evolutions with the maximum nitrate concentrations under different groundwater depths have similar tendencies, and they almost linearly decrease year on year. The nitrate in the saturated region migrates more slowly than that in the unsaturated region, so the migration process can be divided into two main stages. The groundwater depth mainly affects the migration in the unsaturated region, and the whole migration region will decrease as the groundwater depth increases. In the saturated region, the vertical position with the maximum nitrate concentration penetrates by about 0.10 m every year.

## 5. Discussions and Conclusions

The nitrate migration under different conditions has been simulated and analyzed. Because of the rainfall in the vertical direction, nitrate mainly diffuses in the vertical direction, and it slightly diffuses in the horizontal direction. During the migration process, the vertical contaminated range (C > 0.01 mg/mL) first continuously enlarges, and then decreases after a long time. The nitrate migration is remarkably influenced by annual precipitation and soil material. As annual precipitation rises, the nitrate permeates into deeper levels, while the maximum nitrate concentration decreases more quickly. Soils with large percentages of sand have better permeability, and nitrate transport occurs more quickly and deeply. According to the present simulation, this contaminant can exist and affect the environment for as long as 115–625 years, and the nitrate contamination time will be even longer in dry regions or regions with very little sand. Since nitrate diffuses more quickly in unsaturated regions rather than in saturated regions, the whole diffusion region will decrease as the groundwater depth increases.

In this article, the nitrate migration simulation is limited by the assumption of no chemical reactions occurring and the unnatural conditions in which the simulation took place. The associated model and research could be further improved by the inclusion of a chemical reaction and by carrying out the simulation under natural conditions. The nitrate reaction in soil [32,33] includes microbially mediated reactions and/or purely chemical reactions, and it is affected by many factors including the chemical composition of soil, the microbial content of soil, the hydrogeological regime, and many other conditions. In further research, the chemical reaction rate of nitrate should be obtained from an experiment, so that it can act as source sink term *G* in Equation (3). On the other hand, natural conditions should be considered by taking into account the complex soil structure, hydrogeological regime, number of aquifers, presence of hydrogeological windows, ground water flow direction, and so on, and the simulation model or program should be developed for these requirements.

## Figures and Tables

**Table 1:** Characteristics of three soils.

Soil	Sand	Silt	Clay	Density	Porosity	Typical Region
	%	%	%	g/cm^3^	%	
SOIL I	88.65	10.21	1.14	1.472	44.64	Dunhuang
SOIL II	30.08	42.96	26.96	1.64	37.7	Inner Mongolia
SOIL III	28.7	58.26	13.04	1.4	48.16	Beijing

## References

[1] Peiró G., Prieto C., Gasia J., Jové A., Miró L., Cabeza L.F. (2018). Two-tank molten salts thermal energy storage system for solar power plants at pilot plant scale: Lessons learnt and recommendations for its design, start-up and operation. Renew. Energy.

[2] Slessarev I., Bokov P. (2004). Deterministic safety study of advanced molten salt nuclear systems for prospective nuclear power. Nucl. Eng. Des..

[3] Ayub R., Messier K.P., Serre M.L., Mahinthakumar K. (2019). Non-point source evaluation of groundwater nitrate contamination from agriculture under geologic uncertainty. Stoch. Environ. Res. Risk Assess..

[4] Hitchon B., Perkins E.H., Gunter W.D. (1999). Introduction to Ground Water Geochemistry.

[5] Pratiwi E.P.A., Hillary A.K., Fukuda T., Shinogi Y. (2016). The effects of rice husk char on ammonium, nitrate and phosphate retention and leaching in loamy soil. Geoderma.

[6] Leimer S., Oelmann Y., Wirth C., Wilcke W. (2015). Time matters for plant diversity effects on nitrate leaching from temperate grassland. Agric. Ecosyst. Environ..

[7] Kanthle A.K., Lenka N., Lenka S., Tedia K. (2016). Biochar impact on nitrate leaching as influenced by native soil organic carbon in an Inceptisol of central India. Soil Tillage Res..

[8] Liu C.-W., Sung Y., Chen B.-C., Lai H.-Y. (2014). Effects of Nitrogen Fertilizers on the Growth and Nitrate Content of Lettuce (Lactuca sativa L.). Int. J. Environ. Res. Public Health.

[9] Woli P., Hoogenboom G., Alva A. (2016). Simulation of potato yield, nitrate leaching, and profit margins as influenced by irrigation and nitrogen management in different soils and production regions. Agric. Water Manag..

[10] Parrino F., Roda C.-, Loddo V., Palmisano L., Camera-Roda G. (2019). Three-Dimensional Calibration for Routine Analyses of Bromide and Nitrate Ions as Indicators of Groundwater Quality in Coastal Territories. Int. J. Environ. Res. Public Health.

[11] Leslie D.L., Lyons W.B. (2018). Variations in Dissolved Nitrate, Chloride, and Sulfate in Precipitation, Reservoir, and Tap Waters, Columbus, Ohio. Int. J. Environ. Res. Public Health.

[12] Schullehner J., Stayner L., Hansen B. (2017). Nitrate, Nitrite, and Ammonium Variability in Drinking Water Distribution Systems. Int. J. Environ. Res. Public Health.

[13] Loschko M., Wöhling T., Rudolph D.L., Cirpka O.A. (2018). Accounting for the Decreasing Reaction Potential of Heterogeneous Aquifers in a Stochastic Framework of Aquifer-Scale Reactive Transport. Water Resour. Res..

[14] Tafteh A., Sepaskhah A.R. (2012). Application of HYDRUS-1D model for simulating water and nitrate leaching from continuous and alternate furrow irrigated rapeseed and maize fields. Agric. Water Manag..

[15] Jiang S., Pang L., Buchan G.D., Šimůnek J., Noonan M.J., Close M. (2010). Modeling water flow and bacterial transport in undisturbed lysimeters under irrigations of dairy shed effluent and water using HYDRUS-1D. Water Res..

[16] Hlaváčiková H., Novák V., Kostka Z., Danko M., Hlavčo J. (2018). The influence of stony soil properties on water dynamics modeled by the HYDRUS model. J. Hydrol. Hydromech..

[17] Kodešová R., Kapicka A., Lebeda J., Grison H., Kočárek M., Petrovsky E. (2011). Numerical Simulation of Fly-Ash Transport in Three Sands of Different Particle-Size Distributions Using Hydrus-1d. J. Hydrol. Hydromech..

[18] Wu J., Ding J., Lu J. (2016). Nitrate Transport Characteristics in the Soil and Groundwater. Procedia Eng..

[19] Iqbal S., Guber A.K., Khan H.Z. (2016). Estimating nitrogen leaching losses after compost application in furrow irrigated soils of Pakistan using HYDRUS-2D software. Agric. Water Manag..

[20] Shelia V., Šimůnek J., Boote K., Hoogenbooom G. (2018). Coupling DSSAT and HYDRUS-1D for simulations of soil water dynamics in the soil-plant-atmosphere system. J. Hydrol. Hydromech..

[21] Pang L., Close M.E., Watt J.P., Vincent K.W. (2000). Simulation of picloram, atrazine, and simazine leaching through two New Zealand soils and into groundwater using HYDRUS-2D. J. Contam. Hydrol..

[22] Asada K., Eguchi S., Ikeba M., Kato T., Yada S., Nakajima Y., Itahashi S. (2017). Modeling nitrogen leaching from Andosols amended with different composted manures using LEACHM. Nutr. Cycl. Agroecosyst..

[23] Cameira M.D.R., Fernando R., Ahuja L., Ma L. (2007). Using RZWQM to simulate the fate of nitrogen in field soil–crop environment in the Mediterranean region. Agric. Water Manag..

[24] Wu J., Ding J., Lu J., Wang W. (2017). Migration and phase change phenomena and characteristics of molten salt leaked into soil porous system. Int. J. Heat Mass Transf..

[25] Simunek J., Sejna M., Saito H., Sakai M., Genuchten M.T. (2005). The HYDRUS-1D Software Package for Simulating the One-Dimensional Movement of Water, Heat, And Multiple Solutes in Variably-Saturated Media Version 4.0.

[26] Nan H.Y. (2015). Study on community structure of nitrifiers and denitrifiers in soil of different habitats. Master’s Thesis.

[27] Richards L.A. (1931). Capillary conduction of liquids through porous mediums. Physics.

[28] Wu J.Q. (2017). Diffusion and Migration Characteristics of Nitrate Molten Salt after Leakage Processes in the Soil and Groundwater System. Master’s Thesis.

[29] Van Genuchten M.T. (1980). A Closed-form Equation for Predicting the Hydraulic Conductivity of Unsaturated Soils. Soil Sci. Soc. Am. J..

[30] Mualem Y. (1976). A new model for predicting the hydraulic conductivity of unsaturated porous media. Water Resour. Res..

[31] Wang H., Ju X., Wei Y., Li B., Hu K. (2010). Simulation of bromide and nitrate leaching under heavy rainfull and high-intensity irrigation rates in North China Plain. Agr. Water Manag..

[32] Wang M., Hu R., Ruser R., Schmidt C., Kappler A. (2019). Role of Chemodenitrification for N2O Emissions from Nitrate Reduction in Rice Paddy Soils. ACS Earth Space Chem..

[33] Recous S., Mary B., Faurie G. (1990). Microbial immobilizition of ammonium and nitrate in caltivated soils. Soil Biol. Biochem..

